# Discovery of treatment for nerve agents targeting a new metabolic pathway

**DOI:** 10.1007/s00204-020-02820-4

**Published:** 2020-07-27

**Authors:** Trevor Glaros, Elizabeth S. Dhummakupt, Gabrielle M. Rizzo, Ethan McBride, Daniel O. Carmany, Linnzi K. M. Wright, Jeffry S. Forster, Julie A. Renner, Ruth W. Moretz, Russell Dorsey, Mark R. Marten, Walker Huso, Alexander Doan, Carrie D. Dorsey, Christopher Phillips, Bernard Benton, Phillip M. Mach

**Affiliations:** 1 Research and Technology Directorate, BioSciences Division, Combat Capabilities Development Command (CCDC) Chemical Biological Center, 5183 Blackhawk Rd., Building E3150, Aberdeen Proving Ground, Gunpowder, MD 21010 USA; 2 Excet, Inc., 6225 Brandon Ave, Suite 360, Springfield, VA 22150 USA; 3 National Academies of Sciences, Engineering, and Medicine, NRC Research Associateship Programs, 500 Fifth Street, NW, Washington, DC 20001 USA; 4 Research and Technology Directorate, Toxicology and Obscurants Division, Combat Capabilities Development Command (CCDC) Chemical Biological Center, 5183 Blackhawk Rd., Aberdeen Proving Ground, Gunpowder, MD 21010 USA; 5 Kirk U.S. Army Health Clinic, 6455 Machine Rd., Aberdeen Proving Ground, Gunpowder, MD 21005 USA; 6 Department of Chemical, Biochemical and Environmental Engineering, University of Maryland, Baltimore County (UMBC), Engineering Building, Baltimore, MD USA; 7 Present Address: BioSciences Division, B11 Bioenergy and Biome Sciences, Los Alamos National Laboratory, SM30, Mailstop E529, PO Box 1663, Los Alamos, NM 87545 USA

**Keywords:** Proteomics, Metabolomics, Cardiomyocytes, Glycolysis, a-Ketoglutarate, VX poisoning

## Abstract

The inhibition of acetylcholinesterase is regarded as the primary toxic mechanism of action for chemical warfare agents. Recently, there have been numerous reports suggesting that metabolic processes could significantly contribute to toxicity. As such, we applied a multi-omics pipeline to generate a detailed cascade of molecular events temporally occurring in guinea pigs exposed to VX. Proteomic and metabolomic profiling resulted in the identification of several enzymes and metabolic precursors involved in glycolysis and the TCA cycle. All lines of experimental evidence indicated that there was a blockade of the TCA cycle at isocitrate dehydrogenase 2, which converts isocitrate to *α*-ketoglutarate. Using a primary beating cardiomyocyte cell model, we were able to determine that the supplementation of *α*-ketoglutarate subsequently rescued cells from the acute effects of VX poisoning. This study highlights the broad impacts that VX has and how understanding these mechanisms could result in new therapeutics such as *α*-ketoglutarate.

**Electronic supplementary material:**

The online version of this article (10.1007/s00204-020-02820-4) contains supplementary material, which is available to authorized users.

## Introduction

Organophosphates (OPs) represent a wide range of structurally related chemistries that have seen wide spread use as commercial insecticides and, more nefariously, as developed chemical warfare agents (CWAs). These compounds were discovered in the mid-1800s, but their toxic properties were not applied to warfare until the 1930s (Petroianu 2015). Early toxicological studies using tetraethyl pyrophosphate (TEPP), tabun (GA), and sarin (GB) were performed by Germany’s War Ministry and industry. Initial findings revealed that these compounds inhibit cholinesterase, which explained the excessive stimulation of the central nervous system leading to respiratory failure and death. Although nearly 90 years have passed since these early studies, inhibition of acetylcholinesterase (AChE) by OP ‘phosphorylation’ in the enzyme’s catalytic site is still widely accepted as the primary mechanism of action (Maxwell et al. 2006). In the late 1960s, Johnson discovered that delayed neurotoxicity due to OP exposure is initiated by neurotoxic esterase (NTE) inhibition (Johnson 1969). This was perhaps the first evidence suggesting that OPs are more promiscuous than convention suggests and that ‘secondary’ mechanisms of action likely have a significant role in toxicity.

In the late 1990s, Black et al. discovered that OPs not only covalently bind AChE and butyrylcholinesterase (BuChE), but also serum albumin (Black et al. 1999). This study was the first time a CWA was shown to bind to a tyrosine residue and not just serine residues. Additional work has expanded OP binding motifs beyond these residues (Lockridge and Schopfer 2010). Perhaps the most compelling evidence for the lack of OP specificity was work performed by a Dutch team at The Netherlands Organization (TNO). By leveraging a probing strategy known as activity-based protein profiling (ABPP) developed by the Cravatt laboratory (Speers and Cravatt 2004), the Noort group, using two different cell lysates and a liver homogenate, was able to convincingly show that sarin covalently binds to a wide range of proteins (Tuin 2009). Shortly thereafter, Nomura et al. used the ABPP technique to assess the specificity of a wide variety of commonly used OP pesticides and thiocarbamates herbicides, such as chlorpyrifos and tribufos (Nomura and Casida 2011). More recently, our team utilized this ABPP approach for VX; however, in this work, a quantitative proteomics method was leveraged to increase the assay’s sensitivity. By combining these techniques, we were able to demonstrate that VX covalently binds to 132 different proteins, including a wide range of enzymes essential for metabolic processes (Carmany 2017). In this work, mitochondrial isocitrate dehydrogenase (IDH2) was shown to bind covalently to VX, but most importantly, this binding directly inhibited enzymatic activity. This was the first evidence that VX inhibits an essential enzyme for respiration, although it was not the first time VX was implicated in metabolic effects. A team at the United States Army Medical Institute for Chemical Defense (USAMRICD) demonstrated that a ketogenic diet was protective whereas a glucose-enriched diet was detrimental to survival when rats were exposed repeatedly to soman for a month (Langston and Myers 2011; Myers and Langston 2011). Despite this evidence, little is known about the metabolic-linked secondary mechanisms of action.

Treatment of OP poisoning requires early administration of specific antidotes in conjunction with basic life-sustaining care. As respiratory distress and vomiting are among the first symptoms, victim’s airways are cleared and then artificial ventilation is established (Minton and Murray 1988). Effective ventilation of the victim is not possible until sufficient quantities of antidote are administered; therefore, it is critical to administer atropine before or while preparing to establish ventilation. Currently, atropine is the first-line countermeasure as it competitively blocks the action of AChE preventing excessive parasympathetic stimulation. Multiple doses of atropine can be given depending upon the severity of the symptoms. Oximes, most commonly pralidoxime, are co-administered with atropine to assist in ‘reactivating’ acetylcholinesterase. Benzodiazepines are utilized in the event of an OP poisoning, because they cross the blood–brain barrier more effectively than atropine. This treatment counteracts the central nervous system effects of poisoning. Benzodiazepines are critical for the treatment of the seizures resulting from OP poisoning and may prevent seizure onset. A wide range of other medications have been used or explored to alleviate symptoms of poisoning or to directly counteract OP’s known molecular mechanism of action. Prophylactic treatments for at risk populations have been explored including but not limited to: anticonvulsants, anticholinergics, reactivators, calcium antagonists, neuromuscular blockers, and bio-scavengers (Cerasoli 2019; Bajgar 2004; Zhang et al. 2019). With few exceptions, most approaches have proven to be impractical due to timing, duration, and sufficient bioavailability. Thus far, we are unaware of treatment strategies which have been tested to counteract secondary effects of poisoning such as metabolic disruption.

In the last decade, there have been major advances which now allow for the collection of detailed molecular data at the protein, metabolite, and RNA levels; collectively referred to as multi-omics. These technologies have seen large improvements in speed, sensitivity, and accessibility. Importantly, there has also been a concerted effort to develop computational approaches which are capable of interrogating these large data sets both efficiently and accurately. Most recently, there have been many efforts to unify these omic data streams allowing researchers to describe a detailed cascade of molecular events that occur as a result of a particular stimulus. To date, there is an overwhelming amount of evidence which indicates that OPs impact both the proteome and metabolome. Despite this, there are only a few examples of proteomic and metabolomic studies focused on OP CWAs (Meade 2015; Nirujogi 2015).

In the current study, we performed shotgun proteomics and untargeted metabolomics on blood plasma samples obtained from hairless guinea pigs (*N* = 6) which were intravenously exposed to 0.4 LD_50_ VX. By comparing each animal’s plasma prior to exposure to plasma sampled serially over 14 days, we hoped to gain a better understanding of the changes to the types and amounts of endogenous molecules after VX exposure. This approach resulted in a list of proteins which were found to change ± twofold as time points varied in the study. Cytoplasmic isocitrate dehydrogenase (IDH1) was found to be upregulated in all animals by an average of 2.6-fold within the first hour post-exposure. Interestingly, we also observed significantly elevated precursor metabolites for alpha-ketoglutarate (*α*-KG) that precede IDH1 and mitochondrial isocitrate dehydrogenase (IDH2) as well as decreased levels of *α*-KG. Given our previous work, which demonstrated that IDH2 was directly inhibited by VX, it was hypothesized that IDH1 upregulation may be a compensatory mechanism to overcome the VX-induced blockade of the citric acid cycle allowing the cell to generate *α*-KG from isocitrate in the cytosol (Carmany 2017). To determine if this metabolic consequence of VX poisoning could be linked to cellular toxicity, we assessed perturbations to contractility of human-induced pluripotent stem cells (hIPSCs) derived cardiomyocytes to determine if we could ‘rescue’ cells exposed to VX by simply pretreating the culture media with *α*-KG. This pretreatment prevented the immediate effects of VX exposure in a dose-dependent manner. These results strongly suggest that the ‘secondary’ molecular targets of VX intoxication, especially the inhibition of IDH2, may play a critical role in the acute effects of OP poisoning. These findings indicate that *α*-KG may be a suitable countermeasure for OPs when used as a prophylactic or in conjunction with atropine and pralidoxime following exposure. Since *α*-KG is already widely available and already considered ‘generally regarded as safe’ by the FDA, this discovery could result in a new fast-tracked, complementary treatment. With that said, *α*-KG is not an approved therapeutic for OP poisoning and should not be used without first consulting with a medical professional.

## Results

### Proteomic and metabolomic analyses

**Fig. 1:**
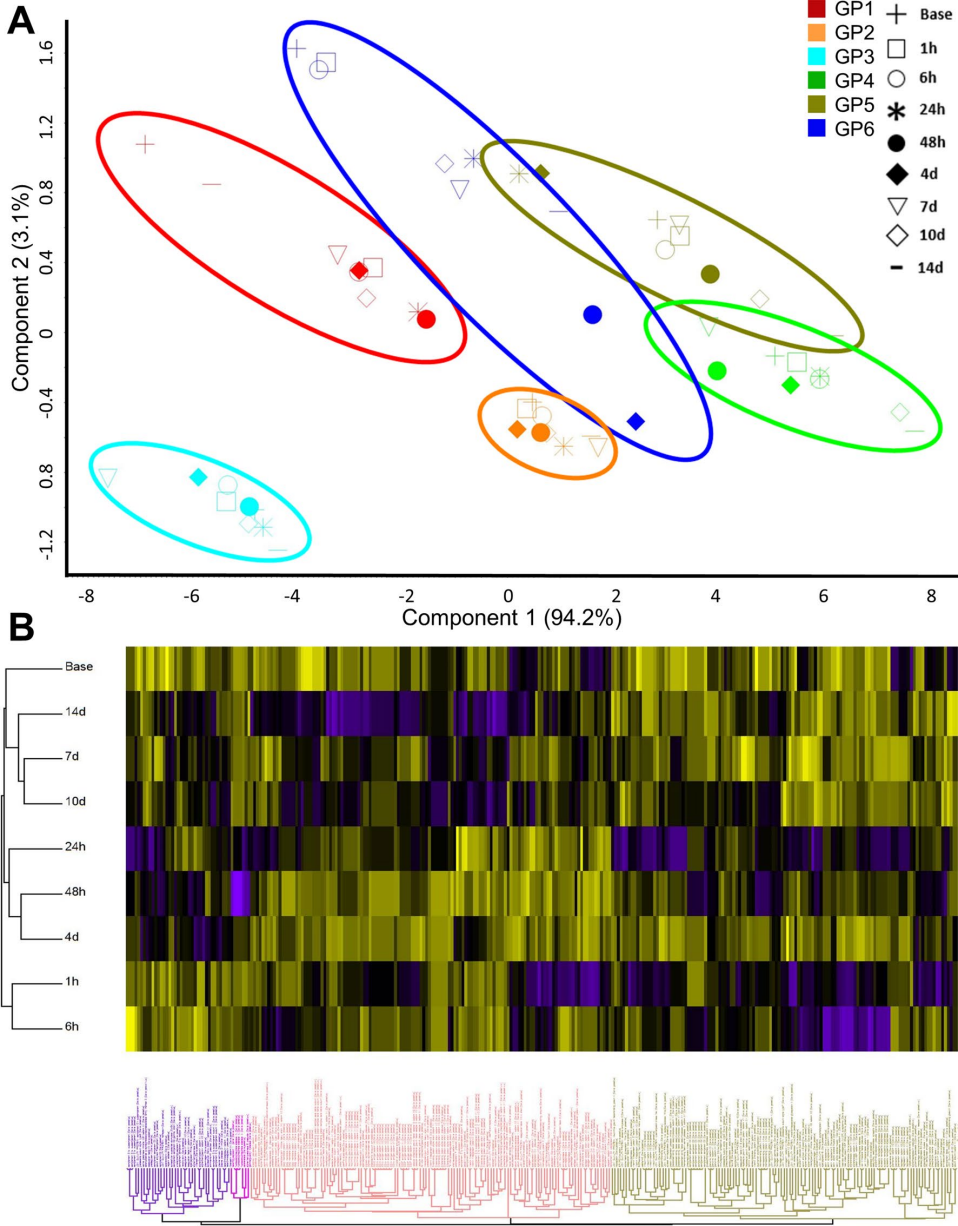
**a** PCA plot of the 295 proteins that change over the duration of the study for each animal. The proteomes cluster by animal and not by time. **b** Hierarchical clustering analysis of 295 proteins also shows that the proteins cluster into four main groups based upon their temporal expression over the 14 day time course

More than 2200 proteins were identified across 6 animals in the study with a minimum report intensity of 5 × 10^3^ in at least eight of the quantitation channels with at least one unique peptide. Reporter intensities were normalized across all animals using the ‘MasterMix’ channel. This allowed for inter-experiment or inter-animal normalization. After normalization across all experimental data, the data were scaled to 100 to calculate relative abundance. Principal component analysis (PCA) and heat map plots of these data show that the samples cluster by animal and not by time post-exposure (Fig. 1). This suggests that there was a great deal of proteome diversity and that the toxicant exposure did not overwhelmingly shift all the animal’s proteomes, consequently, this did not allow for the changes to overcome the noise contributed from the rest of the proteome.

Statistical analysis was performed on the entire proteomic-analysis set using multivariate adaptive regression splines method (MARS) in Matlab (Friedman 1991; Jekabsons 2016). This analysis shows 72 unique proteins demonstrated a statistically significant change in expression over the duration of the study (Table S4). Because we were also interested in magnitude of change, we filtered our data separately by considering only proteins in which expression changed ± twofold in at least four of the six animals. Eighteen proteins showed this level of differential change following exposure to VX. Laminin, hepatocyte growth factor-like protein isoform X1 and X2 were excluded because of a single sample outlier in one animal. Three additional hemoglobin subunits also met our filter criteria, but these proteins sporadically changed throughout the duration of the study. Recent work performed by Geyer et al. attributed these proteins to ‘sample-contamination’, as such, they were disregarded from this study (Geyer et al. 2018). This left 12 unique proteins for which expression changed ± twofold which are listed in Table 1. Of these 12 proteins, 5 also passed the MARS statistical test and are denoted in the final column of Table 1.

**Table 1:**
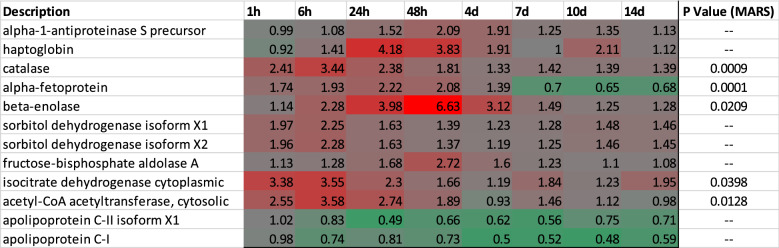
Proteins with ± twofold Change. Unique proteins with ± twofold expression changes. Red indicates an increased expression compared to baseline, and green indicates a decreased expression compared to baseline

**Fig. 2:**
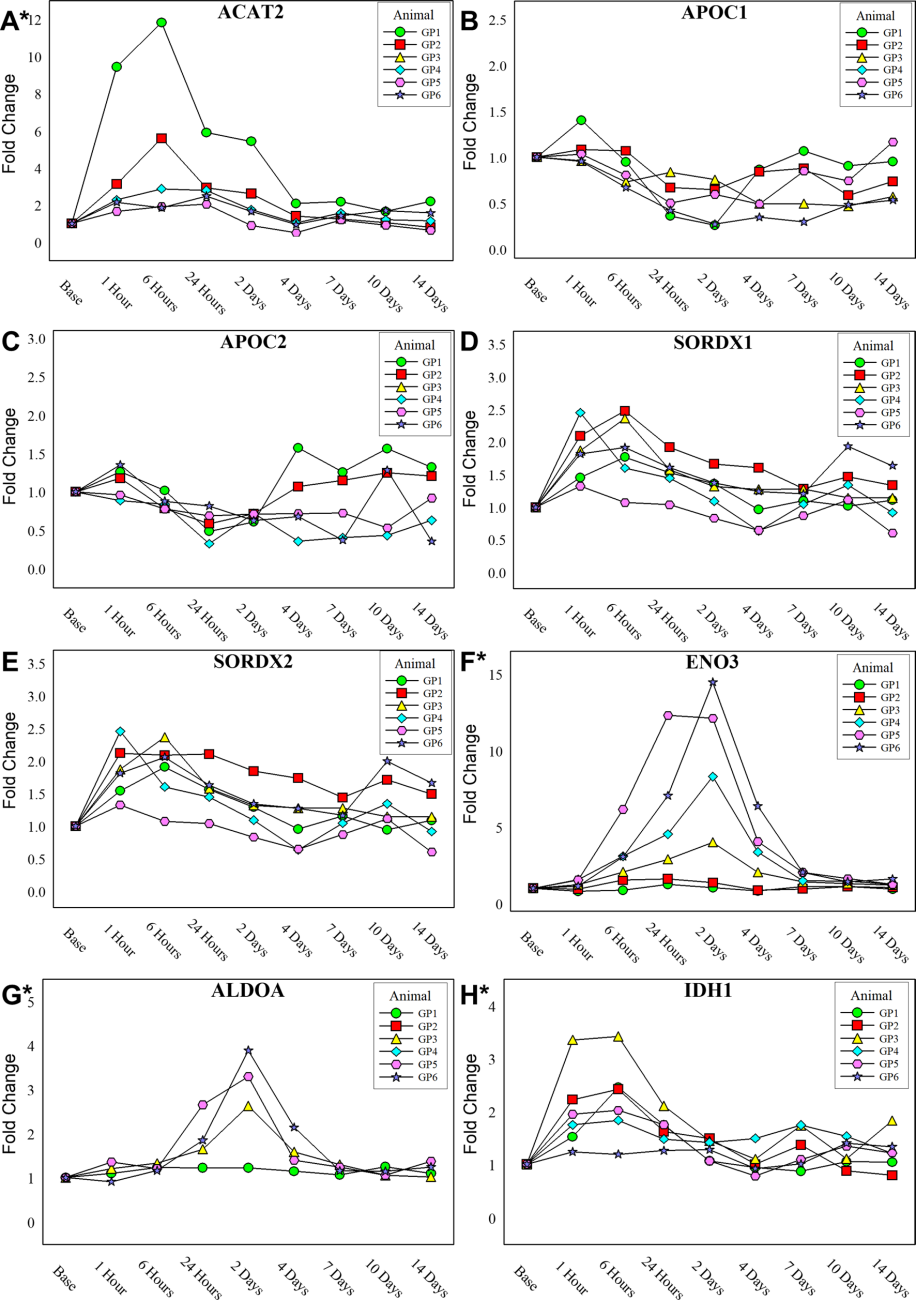
Expression profiles graphed by each individual animal for eight proteins which changed ± twofold and have been shown to be linked to various energy processes. *Indicates statistical significance as determined by MARS analysis

Eight of these 12 proteins (± twofold) are directly involved in energy processes (Fig. 2). Five of these energy-linked proteins are involved in pathways outside of glycolysis and the TCA cycle (Fig. 2a–e). Acetyl-CoA acetyltransferase, cytosolic (ACAT2), apolipoprotein C-II (APOC2), and apolipoprotein C-I (APOC1) are linked to lipid metabolic processes. Interestingly, ACAT2 is significantly upregulated at 1 h, peaks at 6 h, and returns to baseline 4 days post-exposure. APOC2 and APOC1 expression was unchanged 1 h post-exposure, but then steadily decreased throughout the remainder of the study with the low point at 2 days post-exposure. Two isoforms (X1 and X2) of sorbitol dehydrogenase (SORD) were shown to increase within the first hour post-exposure, peak between 1 and 6 h post-exposure, and then return to 1.5-fold increase for the duration of the study. Although this enzyme is not involved in glycolysis, it is responsible for generating d-fructose from d-sorbitol which ultimately gets converted to d-glyceraldehyde 3-phosphate at the fourth step of glycolysis. Beta enolase (ENO3) and fructose bisphosphate aldolase (ALDOA) were both identified and are directly involved in glycolysis (Fig. 2f, g). Each enzyme appears to be upregulated between 6 and 24 h post-exposure and peaks 2 days post-exposure before returning to near baseline sometime between 7 and 10 days.

Finally and perhaps most importantly, we identified IDH1 as being significantly upregulated immediately following exposure to VX (Fig. 2h). The protein on average was upregulated nearly 3.6-fold within 1 h of exposure and remained elevated up to 2 days post-exposure. On the time scale of this study, IDH1 was the first enzyme in either the glycolysis or the TCA cycle to show perturbation. IDH1 is the cytosolic form of isocitrate dehydrogenase which also exists within the mitochondrial matrix (IDH2) where it plays a critical role generating *α*-KG. Using the activity-based protein profiling (ABPP) assay, we were recently able to show that VX directly inhibits IDH2 in a concentration-dependent manner (Carmany 2017). Given that IDH2 is inhibited by VX, the upregulation of IDH1 likely occurs as a means to compensate. Due to the large number of metabolism-linked proteins that were impacted following exposure to VX, next we sought to globally assess the plasma metabolome.

**Fig. 3:**
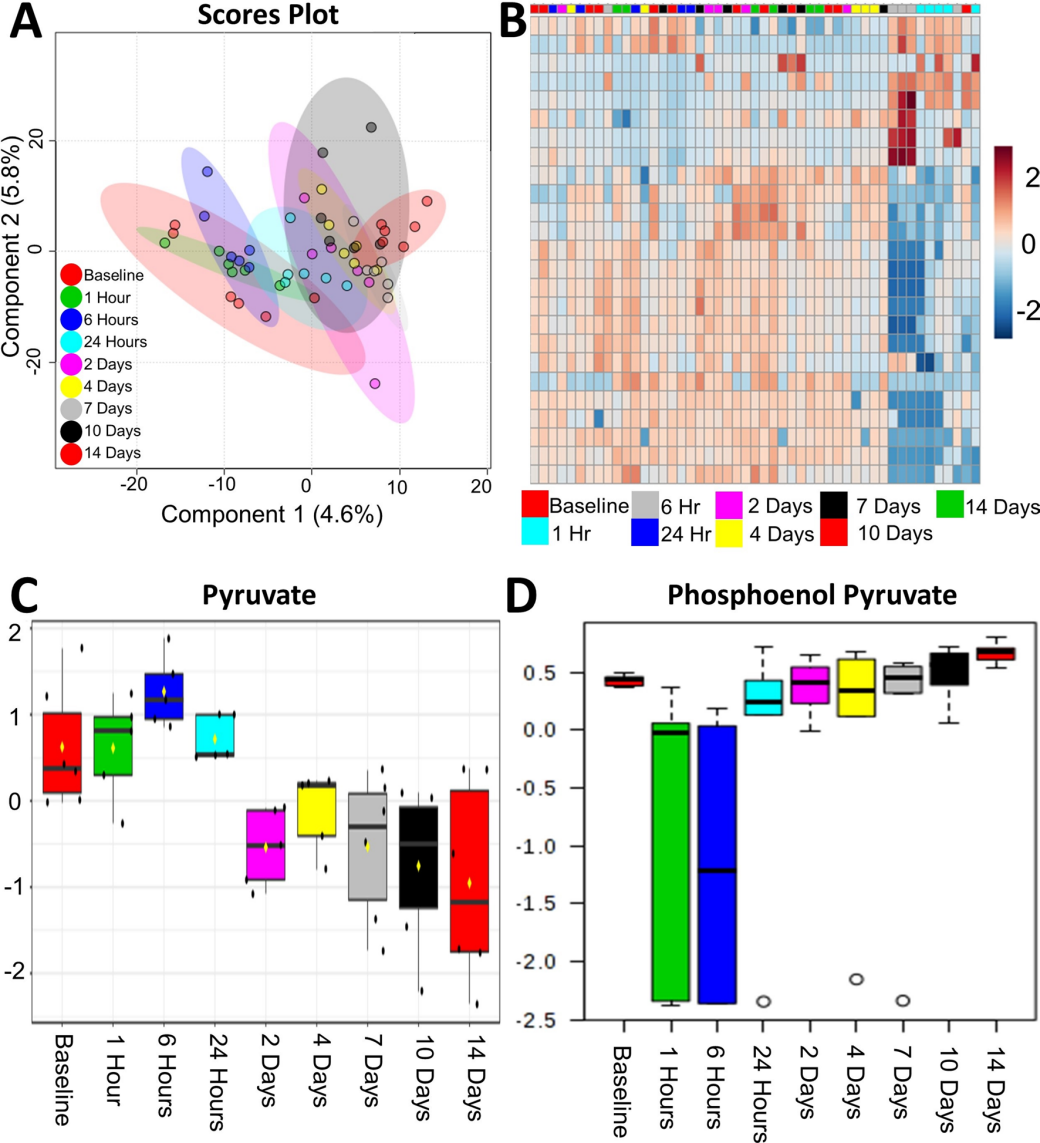
**a** PLS-DA plot of curated metabolite features. Time 0 h, 1 h, and 6 h loosely separate from all other time points post-exposure. **b** Hierarchical clustering analysis also show that the metabolic profiles of the early time points are most similar and distinct from the later time points (right-hand cluster). Box-and-whiskers plots of pyruvate (**c**) and phosphoenol pyruvate (**d**) demonstrating expression profiles over the duration of the experiment

Over 6000 features were curated and 83 unique metabolites were positively identified using an in-house library. Statistical analysis was performed and there was slight separation via partial least squares determinant analysis (PLS-DA) between the baseline, 1 h and 6 h time points from the rest of the time points sampled (Fig. 3a). Several metabolites were identified as ANOVA significant (*p* value < 0.05) (Fig. 3b) in this study. Like the proteomic data, several metabolites from glycolysis and the TCA cycle were identified including pyruvate (Fig. 3c) and phosphoenol pyruvate (Fig. 3d). When considering metabolites for which concentrations were only ± log2-fold changed in at least four time points, more features relating to cellular energy production/metabolism (i.e. glycolysis, pentose phosphate pathway, TCA cycle) were differentially changed after exposure to VX, as seen in Table 2. Several TCA metabolites including pyruvate, citrate/isocitrate, aconitate and oxaloacetate are upregulated during the early time points (≤ 24 h). After 24 h, pyruvate shifts to being downregulated, as well as *α*-KG, and both stay downregulated through the 14-day duration. Succinate and fumarate become slightly downregulated around the 24-h time point, while oxaloacetate is increasingly upregulated from 1 h, peaking at the 6- and 24-h time points, and decreasing slightly through the 14-day time point while maintaining overall upregulation.

**Table 2:**
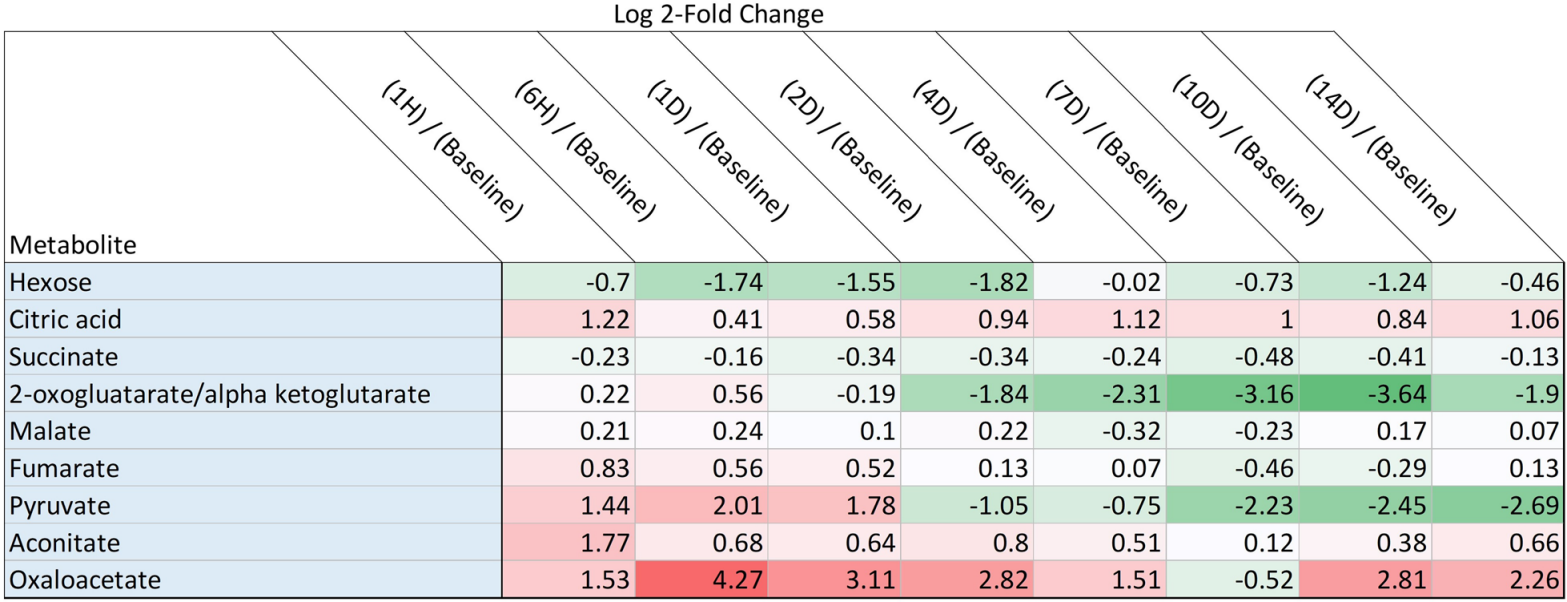
Metabolites with ± twofold Change. Unique metabolites with ± twofold expression changes. Red indicates an increased expression compared to baseline, and green indicates a decreased expression compared to baseline

The dramatic downregulation of *α*-KG between 24- and 48-h indicates a disruption in the TCA cycle (Table 2). As stated previously, VX inhibits IDH2, which is located in the mitochondria and plays a role in *α*-KG synthesis (Carmany 2017). With the inhibition of IDH2, IDH1 is able to compensate by generating *α*-KG from isocitrate in the cytosol which is then transported into the mitochondria allowing for the continuation of the TCA cycle (Harrison and Pierzynowski 2008). The metabolomic results indicated no change in *α*-KG regulation until the 2-day time point, which corroborates the proteomic results. In short, *α*-KG continues to be synthesized while IDH1 is upregulated, but when IDH1 returns to baseline, *α*-KG levels diminish.

Like the proteomic data, additional metabolites involved in other modes of energy generation (i.e. beta-oxidation of fatty acids) were identified as ± log2 fold changed. Fatty acids, like arachidonic acid, oleic acid, docosahexaenoic acid, and linoleic acid, were downregulated from 24-h through 14-days. Several studies of long-term, low-dose exposures to OPs have shown similar metabolic results indicating a dysfunction in glycolysis/TCA cycle and a shift toward beta-oxidation of fatty acids as a response (Bonvallot 2018; Wang 2009; Zhang 2010). Studies performed by Wang et al. investigating serum metabolic profiles of rats exposed to low doses of chlorpyrifos showed decreases in serum lactate levels, which was seen in our study, and a decrease in very low-density lipids (VLDL) (Wang 2009). The two proteins APOC1 and APOC2 are part of the composition of VLDL and were shown to decrease in our study (Fig. 2).

**Fig. 4:**
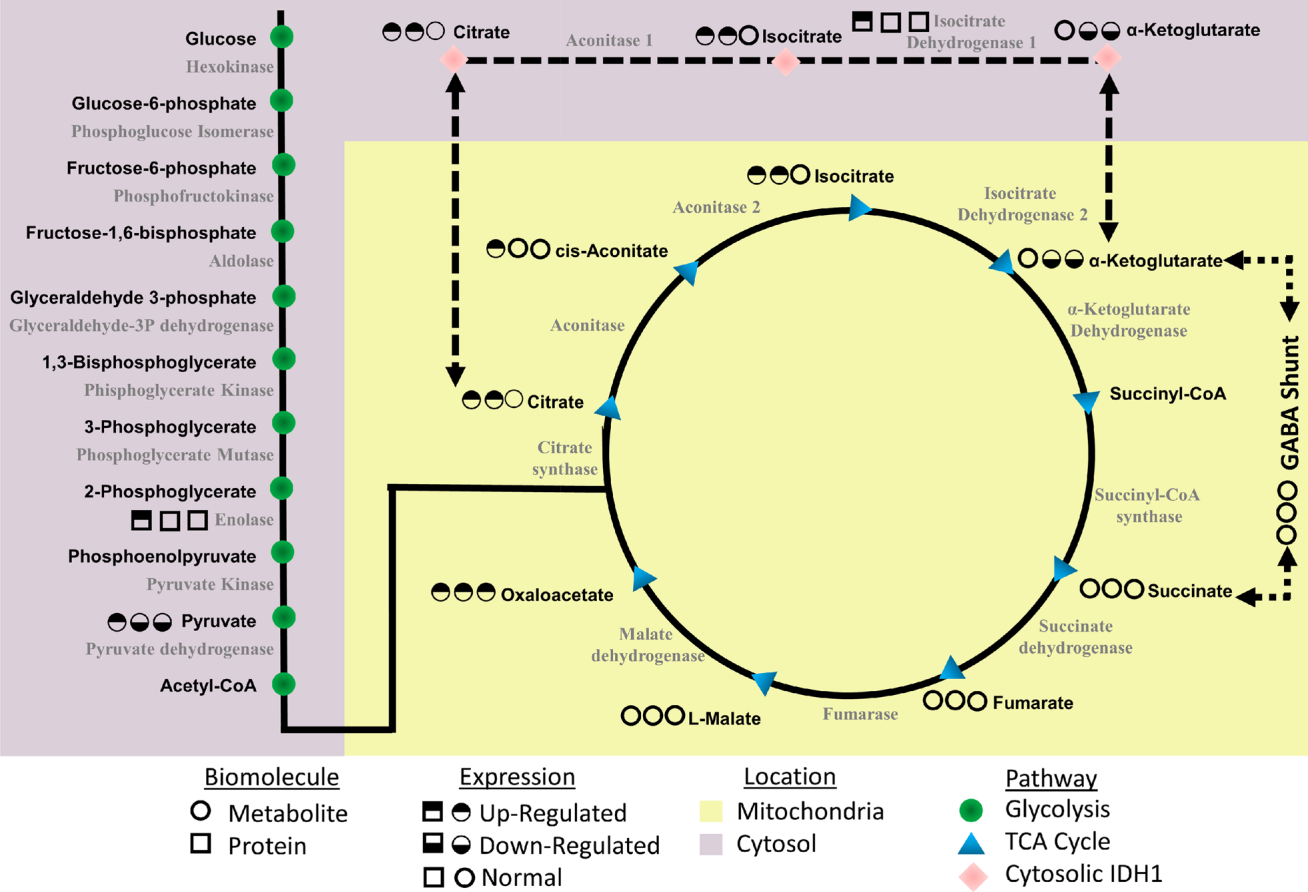
Metabolic and proteomic disruption of glycolysis and the TCA cycle following VX exposure. All proteins and metabolites identified and quantified within these pathways are displayed. A series of symbols next to each protein (square) or metabolite (circle) represent each molecule’s temporal expression profiles. The first circle is a summation of all early time points up to 24 h. The second circle represents all ‘mid’ time points between days 1–7. The final circle denotes all ‘late’ time points between days 7–14

Overall, the proteomic and metabolomic data suggest that exposure to VX leads to an immediate disruption in energy pathways, which for some molecules is long-lasting. As summarized in Fig. 4, there is a dramatic disruption in glycolysis and the TCA cycle as early as 1 h post-exposure, with an intense metabolic downregulation from 24 h onward. This disturbance indicates a shift toward beta-oxidation as an alternative energy source, which is seen in the changes in apolipoproteins and fatty acids (Lei 2008).

### Cardiomyocyte tissue culture modeling for VX poisoning

The development of in vivo models for toxicity have been ongoing for nearly a century. There are numerous examples which show that deriving human toxicity estimates from animals is less than 43% predictive (Olson 2000). This is largely attributed but not limited to species-to-species differences in the P450 isoenzymes. Perhaps some of the most well-known examples include: (1) aspirin which is lethal to cats (Bell 2019) and causes birth defects when paired with caffeine in rodent species (Colomina et al. 2001), (2) penicillin is lethal in guinea pigs, but has no effect in rabbits (Hamre et al. 1943), and (3) morphine is a depressant in humans, but acts as a stimulant in horses (Figueiredo et al. 2012), cats, and goats. Despite these weaknesses, animal models are still used extensively for CWA-related research. Rats, mice, guinea pigs (Harrison et al. 2004), rabbits, ferrets, nonhuman primates (Helden et al. 1983), and swine (Dorandeu 2007) are among the most popular animals used to investigate the effects of a variety of CWAs. From this work, estimates of human toxicity have been derived; however, there remains little evidence as to the accuracy of these numbers due to the lack of human clinical data. Alternative animal models such as zebrafish have also been investigated primarily due to their amenability for high-throughput screening, however these models are still early in development for OPs (Faria 2015). To address these weaknesses, there has been a recent push to develop humanized mouse models to more accurately assess various toxicological mechanisms. For example, a Japanese group recently developed a chimeric mouse model with a humanized liver which produced human hepatic proteins including plasma BuChE that was sensitive to OPs and carbamates (Suemizu et al. 2014, 2018).

**Fig. 5:**
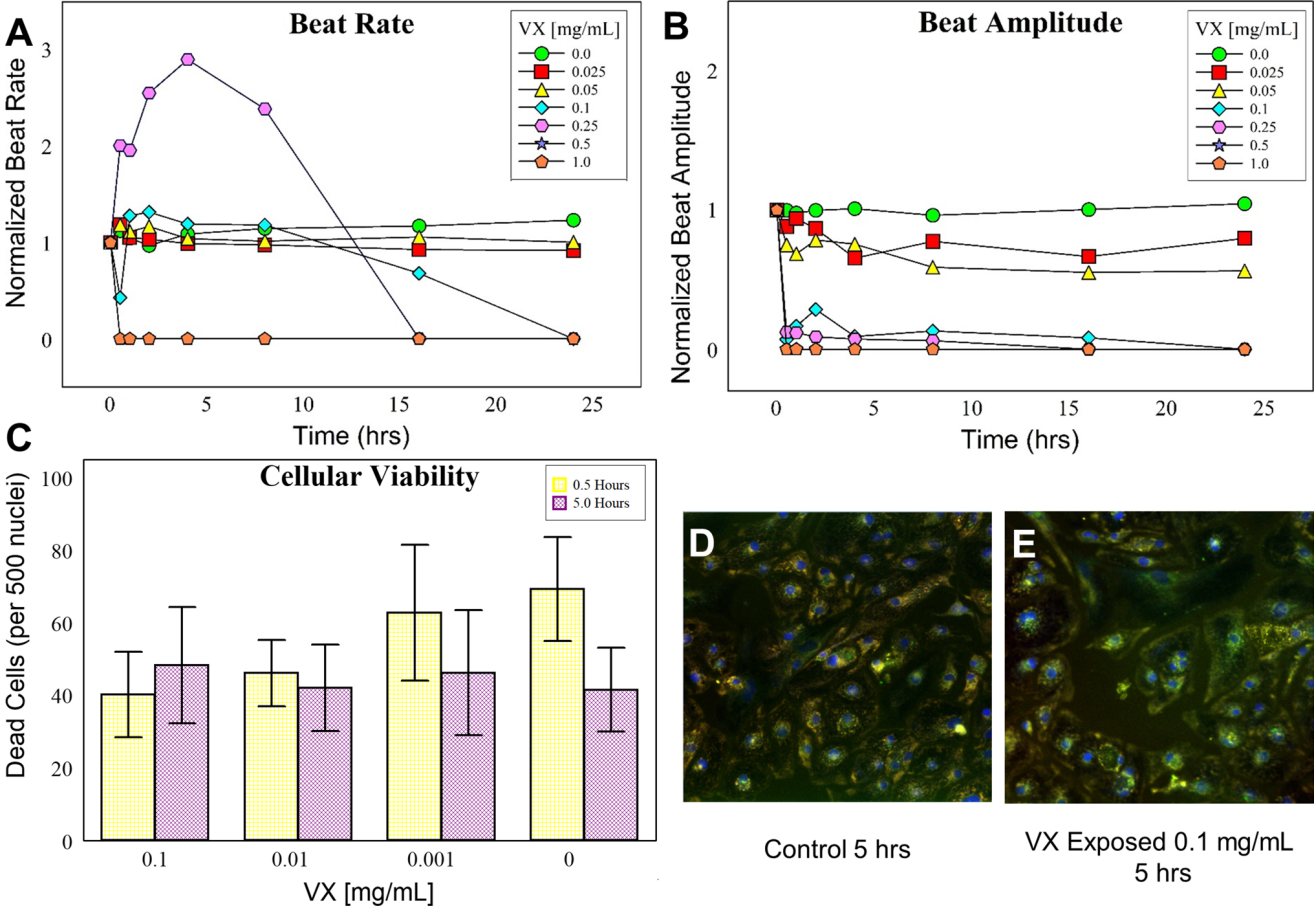
Primary cardiomyocytes as a model for OP exposure. Following varying doses of VX, beat rate (**a**) and beat amplitude (**b**) were assessed. Beat rate and beat amplitude experiments were performed in triplicate, figure images are representative of a single experiment. To ensure these effects were not cytotoxic, cell viability was assessed by high content microscopy. A summation of viability was measured by determining the number of compromised cytoplasmic membranes per 500 nuclei (**c**) over a range of concentrations. These calculation were fully automated by integrating multiple images across a single well of a 96 well tissue culture plate. Representative images from the control (**d**) and exposed at 0.1 mg/mL (**e**) cardiomyocytes are shown

Assessing toxicological effects using in vitro models is much more desirable, especially in regards to efforts to reduce and eliminate animal testing. Most in vitro cell models are unable to simulate what occurs within the living system; in particular, liver metabolism. These metabolic processes can dramatically affect a compound’s toxicity. This is especially true for many OPs including chlorpyrofos, parathion, and malathion. Much less is known about the metabolic processing of OP CWAs in humans following exposure. In fact, nearly all diagnostic methods described in the literature for identifying CWA exposure targets the hydrolyzed alkyl methylphosphonic acid form of each CWA (e.g. VX→Ethyl methylphosphonic acid, GB→Isopropyl methylphosphonic acid), which readily form during environmental degradation. New, more complex cell culturing systems collectively known as microphysiological systems (MPS) aim to address these short comings, especially for generating toxicology estimates and for candidate drug screening. Most OPs do not exhibit cytotoxic effects in cell culture models. Therefore, measuring well-established endpoints such as mitochondrial reductase activity (MTT assay) and apoptosis have little utility for OP CWAs. OPs are known to cause cardiac abnormalities in nearly two-thirds of poisoned patients (Peter et al. 2014). Cardiac effects most commonly manifest as arrhythmias including QTc prolongation (Ludomirsky 1982), ST-T segment changes (Taira et al. 2006), and T wave abnormalities (Karki et al. 2004). This prior evidence in combination with the rapid increase in circulating beta enolase, a known marker for cardiac damage, formed the rationale to explore a cardiac cellular model to study VX’s toxicity. By leveraging Acea Biosciences’s xCELLigence system in conjunction with hIPSCs we established a physiological cell culture model for detecting the cardio-specific consequences of VX poisoning. As shown in Fig. 5a, doses of VX greater than or equal to 0.1 mg/mL cause the cardiomyocytes to stop beating within 24 h of exposure. At doses greater than 0.5 mg/mL, the cellular beating is immediately halted. Interestingly, at 0.25 mg/mL, we noticed an increased beat rate for the first 12 h post-exposure followed by a cessation of beating by 16 h. Beat amplitude or strength showed a similar pattern, however, this parameter appeared to be more sensitive to VX (Fig. 5b). At this particular dose, the cells appear to be ‘mildly’ symptomatic as an effect is measurable, but not catastrophic halting cellular beating. Obvious differences are easily ascertained even at the lowest dose used (25 µg/mL). To confirm that these effects are not due to cell death, high content microscopy was performed to measure plasma membrane integrity and intracellular esterase activity. As shown in Fig. 5c–e, our results also show that VX does not have cytotoxic properties in this primary human cardiomyocyte model.

### α-KG pretreatment prevents acute toxicity in vitro

**Fig. 6:**
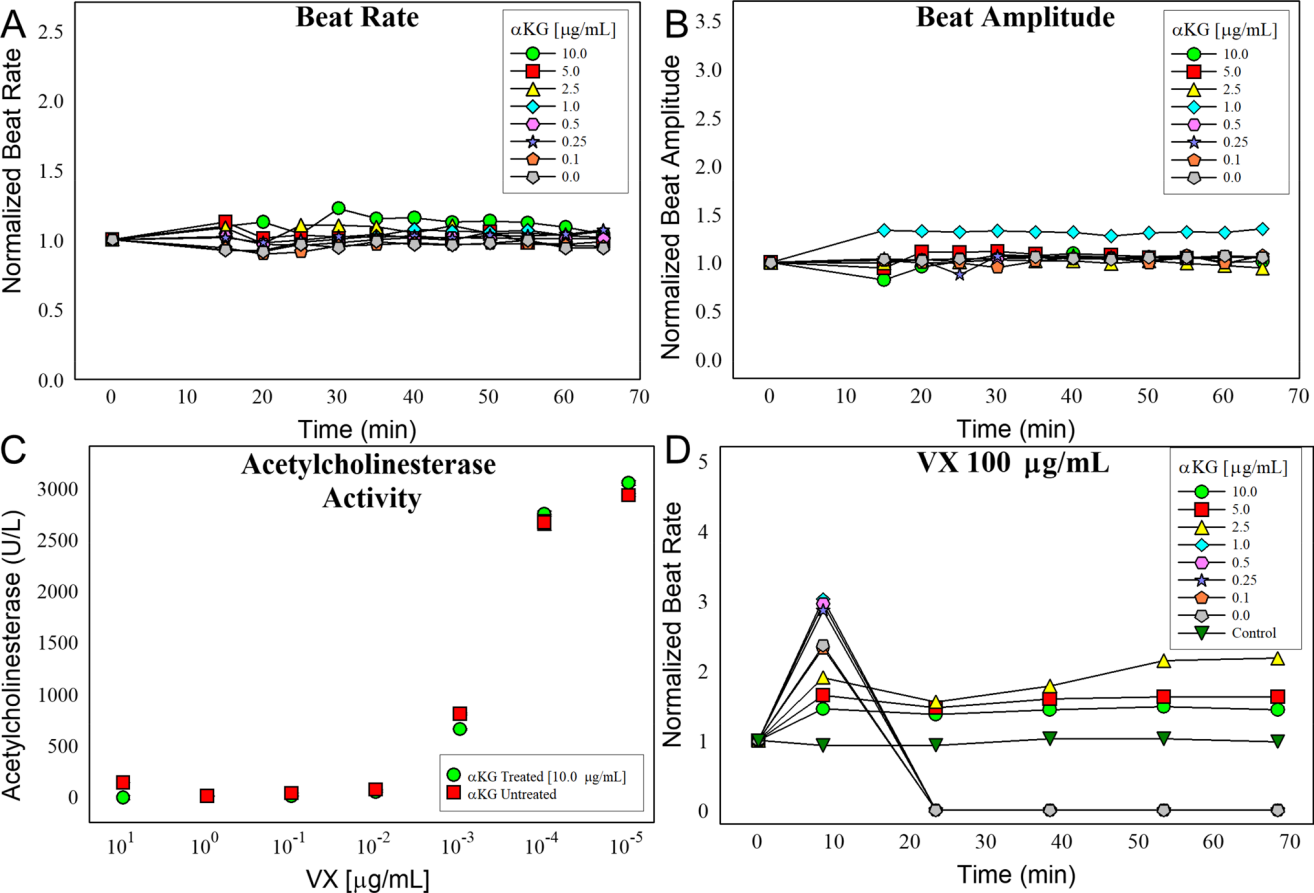
Establishing the effects of *α*-KG on primary cardiomyocytes. Beat rate (**a**) and beat amplitude (**b**) was measured following a 1 h pretreatment of *α*-KG at concentrations ranging between 0.1 and 10 µg/mL. **c** Ellman’s assay of VX exposed blood, with and without *α*-KG to determine if *α*-KG effects AChE inhibition. **d** Beat rate of cardiomyocytes which were pretreated with *α*-KG at varying concentrations for 1 h and then exposed to 100 µg/mL of VX over 70 min. Beat rate and beat amplitude experiments were performed in triplicate, figure images are representative of a single experiment

Based on our multi-omic evidence which implies that VX causes an energy crisis by directly inhibiting mitochondrial IDH2, we hypothesized that we may be able to ‘rescue’ cells from the acute effects of VX poisoning by providing them with excess *α*-KG. Supplementation of *α*-KG would allow for the continued generation of energy via cellular respiration despite the inhibition of IDH2. To test this hypothesis, we first needed to establish if the supplementation of *α*-KG to the culture media would impact the highly sensitive, beating human cardiomyocytes. As shown in Fig. 6a and b, the addition of up to 10 µg/mL of *α*-KG did not change the beat rate or the beat strength in the system. Since AChE inhibition is thought to be the primary mechanism of action from OP poisoning, we wanted to insure that *α*-KG did not prevent the inhibition of AChE upon OP exposure. To test this, we exposed human blood to varying concentration of VX with and without *α*-KG and measured the change in activity of AChE. As shown in Fig. 6c, *α*-KG did not prevent the inhibition of AChE.

**Fig. 7:**
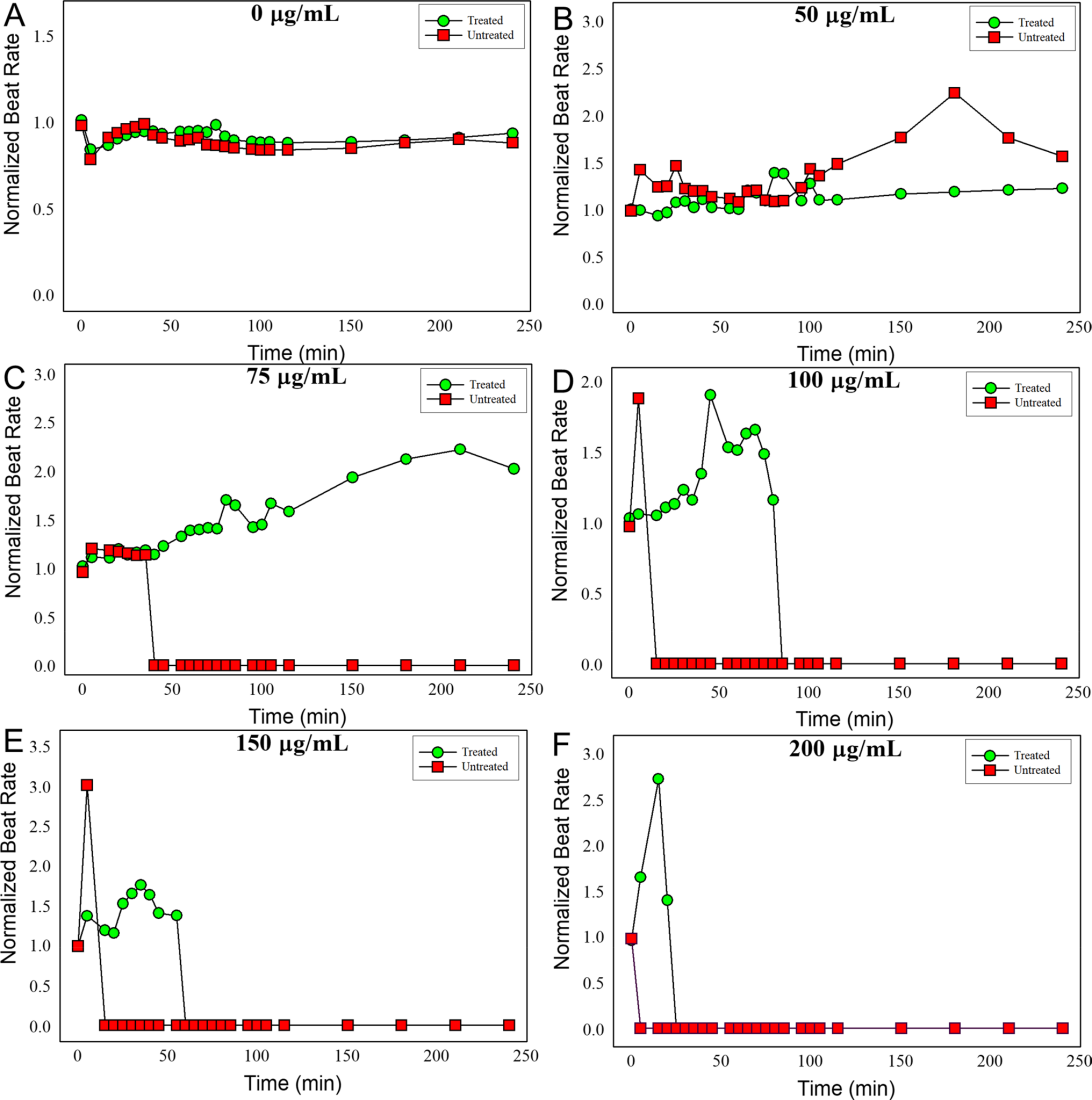
*α*-KG ‘rescues’ primary cardiomyocytes from the acute effects of VX poisoning. Cells were pretreated with 10 µg/mL of *α*-KG for 1 h and then challenged with increasing concentrations of VX. Normalized beat rate for all concentrations was measured for 240 min post-exposure (**a**–**f**). Beat rate and beat amplitude experiments were performed in triplicate, figure images are representative of a single experiment

To determine if we could rescue the cells from the acute effects of VX, we pretreated cells with varying *α*-KG concentrations 1 h prior to exposure with VX at 100 µg/mL (Fig. 6d). This experiment resulted in an *α*-KG dose-dependent decrease in the normalized beat rate elevation following VX exposure. When *α*-KG was used at concentrations below 2.5 µg/mL the cardiomyocytes all stopped beating within the first 25 min post-exposure. At concentrations above 2.5 µg/mL, the cells still had an elevated beat rate through the duration of the study, and remained beating for at least 1 h post-exposure. Since *α*-KG had an additive effect and did not impact the cells at 10 µg/mL, we fixed the *α*-KG concentration and varied the VX concentration (Fig. 7). At all VX concentrations greater than or equal to 75 µg/mL, the addition of α-KG preserved beat rhythm and amplitude. However, with the exception of 75 µg/mL, the cardiomyocytes all eventually stopped beating in a time/dose-dependent manner. In other words, as the dose of VX increased, the time it would take for the cells to stop beating steadily decreased. At the high dose tested (200 µg/mL), the cells stopped beating within the first 5 min, but the presence of α-KG allowed the cells to continue beating for up to 25 min (Fig. 7f).

## Discussion

Collectively, this work demonstrates that *α*-KG ameliorates the toxic effects of VX in cardiomyocytes; however, via a mechanism unrelated to the inhibition of AChE. It is plausible that more commonly described properties of *α*-KG are responsible. For example, *α*-KG is known to protect against oxidative stress (Zdzisińska et al. 2017). In fact, *α*-KG has been shown to protect against cyanide poisoning both in vitro and in vivo* (*Satpute et al. 2010; Bhattacharya et al. 2002). Like OPs, cyanide is also a neurotoxin which causes oxidative stress by the inhibition of numerous antioxidant enzymes (Müller and Krieglstein 1995; Ardelt et al. 1994; Solomonson 1983). Similar to the data presented here, cyanide also disrupts mitochondrial activity preventing cellular respiration by inducing anaerobic metabolism and ultimately causing cell death (Hariharakrishnan et al. 2009). The similarities in OP and cyanide poisoning are remarkable, especially in terms of these chemistry’s impact on mitochondrial respiration. In fact, Holmuhamedov et al. demonstrated that the OP ethaphos disrupted normal mitochondrial processes but not by compromising inner membrane integrity or ATPase functionality (Holmuhamedov et al. 1996). Interestingly, the addition of ATP-rescued mitochondria from the effects of ethaphos poisoning suggesting that an energy crisis is to blame. Similar results have also been shown in neuronal cell culture following exposure to OPs (phenyl saligenin phosphate and mipafox) that cause OP-induced delayed neuropathy (OPIDN) (Massicotte et al. 2005). As an aside, in this publication, the authors state that “mitochondria are an important early target for OP compounds”. Given this evidence and the finding that *α*-KG rescues cardiomyocytes from the acute effects of OP poisoning, it appears that the inhibition of IDH2 may be central to the non-cholinergic toxicity of VX and potentially a wide range of other OPs.

## Online methods

### Caution

Due to the acute hazards with VX, all experiments involving VX were performed by qualified personnel in certified chemical fume hoods equipped with an advanced filtration system that protects the user and the environment at the Combat Capabilities Development Command (CCDC) Chemical Biological Center (Edgewood, MD, USA) according to all Federal, State, and International guidelines.

### Reagents

VX (90.8 ± 0.8% pure as determined by 31P nuclear magnetic resonance spectroscopy) was synthesized at CCDC CBC. Adjusting for purity, a 1 mg/mL stock solution was prepared in normal saline and stored at − 20 °C for the duration of the experiment. The stock solution was then diluted to the appropriate concentration with saline on the mornings of exposure, and these concentrations were verified using gas chromatography–mass spectrometry. Unless otherwise noted, all reagents were obtained from Sigma Aldrich (St. Louis, MO, USA).

### Animal exposures

While conducting research involving animals, the investigators adhered to the current edition of the Guide for the Care and Use of Laboratory Animals. This research was also performed in accordance with the requirements of AR 70-18, The Use of Animals in DoD Programs (Laboratory Animals, Procurement, Transportation, Use, Care and Public Affairs), and the Institutional Animal Care and Use Committee, which oversees the use of laboratory animals by reviewing for approval all animal-related protocols at CCDC CBC. CCDC CBC is fully accredited by AAALAC International.

IAF hairless guinea pigs (male; body weight, 300–350 g) surgically implanted with double jugular vein catheters connected to PinPorts (Instech Laboratories, Inc.; Plymouth Meeting, PA, USA) were purchased from Charles River Laboratories International, Inc. (Kingston, NY, USA). Hairless guinea pigs were single-housed in a temperature- and humidity-controlled colony room (22 ± 4 °C and 55 ± 15%, respectively) with the lights on from 0600 to 1800. Food and water were provided ad libitum in home cages where the hairless guinea pigs also had access to enrichment items. Hairless guinea pigs were acclimated to the facility for at least 4 days prior to exposure. Six hairless guinea pigs weighing 335 ± 26 g were intravenously exposed to 0.4 × LD_50_ VX (4.8 µg/kg) using the left jugular vein catheter. Toxic signs were monitored and recorded continuously for the first 15 min post-exposure followed by intermittent recording at 15-min intervals until 2 h post-exposure and then 1-h intervals until 6 h post-exposure (Table S3). Toxic signs were recorded one final time at 24 h post-exposure. Repeated blood samples were collected as described below. All guinea pigs were euthanized at 14 days post-exposure with the intravenous administration of a barbiturate euthanasia solution.

### Blood collection

Using the right jugular vein catheter, repeated blood samples were collected from each hairless guinea pig intravenously exposed to 0.4 LD50 VX at 24 h pre-exposure (control), as well as 1 h, 6 h, 24 h, 48 h, 4 days, 7 days, 10 days and 14 days post-exposure. The total volume collected in a 24-h period did not exceed 1% of the total blood volume for a hairless guinea pig (~ 250 µL). After each blood draw, catheters were flushed with normal saline and locked with TCS catheter lock solution (Access Technologies; Skokie, IL, USA). Blood samples were transferred to K2 EDTA Micro500 blood collection microtubes (SAI Infusion Technologies; Lake Villa, IL, USA). The tubes were inverted three times to distribute the anticoagulant and then placed on ice. Plasma separation was achieved by spinning collection tubes at 2500 × *g* for 20 min at 4 °C. Plasma was then removed from the tubes and aliquoted prior to storage at − 80 °C.

### Proteomics

See supplemental information for full method. Briefly, a 150 µg aliquot of each plasma sample was subjected to overnight in-solution trypsin/Lys-C digestion. Following digestion, samples were desalted, dried down, and resuspended to a concentration of 2 mg/mL. A “MasterMix” pool was then created by combining 3 µL of each of the 54 samples. Peptide quantification was then carried out on all samples, including the MasterMix, and 30 µg of each sample was removed (with six 30 µg aliquots removed from the MasterMix) to perform TMT labeling. Each animal was labeled using the same TMT kit and labeling scheme for a total of six-10plexes, with each 10plex including one of the identical MasterMix aliquots. Samples were then pooled together based on animal, with one MasterMix per pool, for a total of six TMT plexes. Each pool was subjected to basic reverse phase liquid chromatography (bRPLC) using a 65 min gradient ranging from 0% B to 60% B. Fractions were concatenated into three “start” fractions, five “end” fractions, and 14 mid-phase fractions (designated F1–F14). The fractions were acidified to ~ pH 3, dried, and resuspended with the three “start” and five “end” fractions being combined into respective “start” and “end” vials. Fractions were then subjected to LC–MS/MS analysis on a Thermo Fisher Q Exactive Plus using 2 µL injections resolved on a 75 µm × 50 cm EASY-Spray column using a 182 min multistep gradient ranging from 0% B to 90% B. Spectral data were analyzed using PEAKS X software (Bioinformatics Solutions Inc., Waterloo, ON, Canada). For quantification, inter-experiment normalization was performed using the − 131 MasterMix channel as the spiked channel, with auto normalization selected. Results were filtered to include proteins with at least 1 unique peptide and a fold-change of 2 or greater.

### Metabolomics

See supplemental information for full method. Briefly, samples were prepared via the method detailed in McBride et al. 50 μL of plasma was mixed with 410 μL of extraction solution (8:1:1 acetonitrile:methanol:acetone) containing an isotopically labeled internal standard mixture (McBride 2019). Each sample was vortexed and incubated at 4 °C for 60 min, then centrifuged at 20,000 × *g* for 10 min at 4 °C in order to pellet the precipitate. A 375 μL aliquot of supernatant was transferred to a new centrifuge tube and fully dried, then stored at − 80 °C until analysis. Prior to analysis, samples were reconstituted with 50 μL water with 0.1% formic acid, briefly vortexed, and transferred to glass autosampler vials. Samples were analyzed by reverse phase ultra-high pressure liquid chromatography mass spectrometry (RP-uHPLC-MS) utilizing a Thermo Fisher Ultimate 3000 analytical system coupled to a Thermo Fisher Orbitrap Q Exactive Plus. An ACE Excel 1.7 C18-PFP column (Mac-Mod Analytical; 100 mm × 2.1 mm id) was used for LC separation. Data were collected in positive and negative modes and analyzed by MZmine 2.3 (Pluskal et al. 2010), Metaboanalyst (Version 4, https://www.metaboanalyst.ca), and Compound Discoverer 3.1 (Thermo Fisher Scientific, San Jose, CA, USA).

### Cell culture

Fibronectin was diluted to 10 µg/mL with sterile Dulbecco’s PBS (Gibco, Thermo Fisher Scientific, Waltham, MA, USA) and 50 μL was used to coat the inner wells of a E-plate Cardio 96 (Cat# 06417051001, Acea Biosciences, Inc., San Diego, CA, USA). The plates were incubated at 4 °C overnight. The fibronectin solution was removed and the wells were rinsed once with 200 µL DPBS. Background impedance was recorded using 50 µL of iCell^®^ maintenance medium (Cat# M1003, Cellular Dynamics International, Madison, WI, USA). Cardiomyocytes (Cat#C1056, Cellular Dynamics International) were thawed for 4 min in a 37 °C water bath and transferred to room temperature plating medium (Cat# M1001, Cellular Dynamics International) to a final concentration of 6 × 10^5^ cells/mL. The cells were seeded at 30,000 cells per well. The cells were allowed to settle undisturbed for 30 min at room temperature, and then incubated at 37 °C with 5% CO_2_. After 2 days, the media was changed to maintenance medium. After 4 days, the cells were checked to determine if a consistent, stable, and synchronous beating pattern was established. *α*-KG was prepared to 1 mg/mL and pH adjusted with sodium bicarbonate to pH 7.4. Prior to testing, the *α*-KG was diluted into fresh Roswell Park Memorial Institute (RMPI) 1640 (Gibco, Thermo Fisher Scientific) with B27 supplement (Cat# A3582801, Thermo Fisher Scientific). On the day of testing, VX was prepared by diluting neat material directly into DMEM/B27 to a concentration of 1 mg/mL and then diluted as required. Just prior to testing, the maintenance medium was replaced with 90 µL of fresh DMEM/B27 before taking *T*_0_ reading and returning cells to the incubator. After the cells returned to a consistent, stable, and synchronous beating pattern, the *T*_0_ reading was recorded. 10 µL of *α*-KG at a 10X working concentration was then added and the plate was returned to the instrument. After 30 min, 20 µL of VX at a 5X working concentration was added to the cells. The cells were returned to the instrument and the beat patterns were recorded at the desired time points. The total impedance and each of the beating parameters were recorded for every sweep by the RTCA Cardio software (Version 1.0.1.1203). The beat rates were normalized to the rate just prior to the addition of VX. Total impedance and beating characteristics vary between each well, and as such, every well was treated as an independent unit.

### High content automated microscopy screening

Cardiomyocytes were prepared as described above with the following changes. On the day of testing, VX was prepared by diluting neat material directly into DMEM/B27 to a concentration of 1.0, 0.1, and 0.01 mg/mL. Prior to testing, the maintenance medium was replaced with 90 µL of fresh DMEM with B27. After 2 h, 10 µL of VX was added to the cells. After 5 h of exposure, cells were washed and then incubated for 1 h with five different fluorescence probes (Molecular Probes, USA): Hoechst 33258 to stain nucleic acids, fluo-4-acetoxymethyl ester (Fluo-4AM) to monitor cytosolic free calcium, tetramethylrhodamine methyl ester (TMRM) to assess mitochondrial membrane potential, and green-fluorescent calcein-AM to indicate intracellular esterase activity, and red-fluorescent ethidium homodimer-1 to indicate loss of plasma membrane integrity. Fluorescence channel 1 was utilized for Hoechst 33258 which labelled cellular nuclei to define a primary cellular object in fluorescence, Channel 2 was utilized for calcium, Channel 3 was utilized for mitochondrial integrity, and Channel 4 for the cell viability. Targets were measured using the circ (nuclear) region and the area measurement was expanded for the whole cell. For image analysis, the 20 × objective was used to collect at least 500 cells for each fluorescence channel. Fluorescence images were acquired using the Cellomics ArrayScan VTI HCS Reader (Thermo Scientific, USA) and appropriate filter settings. Fluorescence intensity was analyzed using HCS Navigator Software 6.6.0.

### Acetylcholinesterase activity

Enzymatic activity for AChE was tested using a variation of the Ellman assay (Ellman et al. 1961; Worek et al. 1999). Briefly, whole human blood was exposed to concentrations of VX with and without 10 µg/mL *α*-KG, while being mixed at 37 °C. They were combined with a 2X master mix of buffer, substrate, and developer. All available cholinesterases in the blood hydrolyzed the substrate generating thiocholine. The thiocholine reacted with the developer to generate 5-thio-2-nitrobenzoate anion. The anion was measured at an absorbance of 405 nm. The colorimetric change was plotted over time to gauge the enzymatic inhibitory activity of the samples.

## Electronic supplementary material

Below is the link to the electronic supplementary material.

Supplementary file1 (DOCX 473 kb)

## References

[CR1] Ardelt B, Borowitz J, Maduh E, Swain S, Isom G (1994). Cyanide-induced lipid peroxidation in different organs: subcellular distribution and hydroperoxide generation in neuronal cells. Toxicology.

[CR2] Bajgar J, Makowski GS (2004). Organophosphates/nerve agent poisoning: mechanism of action, diagnosis, prophylaxis, and treatment. Advances in clinical chemistry.

[CR3] Bell J (2019). Aspirin killed the cat: animal research models do not always apply to humans. Exp Opin Drug Metabol Toxicol.

[CR4] Bhattacharya R, Rao PL, Vijayaraghavan R (2002). In vitro and in vivo attenuation of experimental cyanide poisoning by α-ketoglutarate. Toxicol Lett.

[CR5] Black RM, Harrison JM, Read RW (1999). The interaction of sarin and soman with plasma proteins: the identification of a novel phosphonylation site. Arch Toxicol.

[CR6] Bonvallot N (2018). Metabolome disruption of pregnant rats and their offspring resulting from repeated exposure to a pesticide mixture representative of environmental contamination in Brittany. PLoS ONE.

[CR7] Carmany D (2017). Activity based protein profiling leads to identification of novel protein targets for nerve agent VX. Chem Res Toxicol.

[CR8] Cerasoli DM (2019). Butyrylcholinesterase, a stereospecific in vivo bioscavenger against nerve agent intoxication. Biochem Pharmacol.

[CR9] Colomina MT, Albina ML, Sanchez DJ, Domingo JL (2001). Interactions in developmental toxicology: combined action of restraint stress, caffeine, and aspirin in pregnant mice. Teratology.

[CR10] Dorandeu F (2007). Swine models in the design of more effective medical countermeasures against organophosphorus poisoning. Toxicology.

[CR11] Ellman GL, Courtney KD, Andres V, Featherstone RM (1961). A new and rapid colorimetric determination of acetylcholinesterase activity. Biochem Pharmacol.

[CR12] Faria M (2015). Zebrafish models for human acute organophosphorus poisoning. Sci Rep.

[CR13] Figueiredo JP, Muir WW, Sams R (2012). Cardiorespiratory, gastrointestinal, and analgesic effects of morphine sulfate in conscious healthy horses. Am J Vet Res.

[CR14] Friedman JH (1991). Multivariate adaptive regression splines. Ann Stat.

[CR15] Geyer PE et al. (2018) Plasma proteome profiling to detect and avoid sample-related biases in biomarker studies. bioRxiv 478305 10.15252/emmm.201910427 PMC6835559 31566909

[CR16] Hamre DM, Rake G, McKee CM, Macphillamy HB (1943). The toxicity of penicillin as prepared for clinical use. Am J Med Sci.

[CR17] Hariharakrishnan J, Satpute RM, Prasad GBKS, Bhattacharya R (2009). Oxidative stress mediated cytotoxicity of cyanide in LLC-MK2 cells and its attenuation by alpha-ketoglutarate and *N*-acetyl cysteine. Toxicol Lett.

[CR18] Harrison AP, Pierzynowski S (2008). Biological effects of 2-oxoglutarate with particular emphasis on the regulation of protein, mineral and lipid absorption/metabolism, muscle performance, kidney function, bone formation and cancerogenesis, all viewed from a healthy ageing perspective state of the art-review article. J Physiol Pharmacol.

[CR19] Harrison PK, Sheridan RD, Green AC, Scott IR, Tattersall JE (2004). A guinea pig hippocampal slice model of organophosphate-induced seizure activity. J Pharmacol Exp Ther.

[CR20] Holmuhamedov EL, Kholmoukhamedova GL, Baimuradov TB (1996). Non-cholinergic toxicity of organophosphates in mammals: interaction of ethaphos with mitochondrial functions. J Appl Toxicol.

[CR21] Jekabsons G (2016) ARESLab: adaptive regression splines toolbox for Matlab/Octave (ver. 1.10. 3). Institute of Applied Computer Systems Riga Technical University, Latvia. https://www.cs.rtu.lv/jekabsons/Files/ARESLab.pdf. Accessed 20 Apr 2020

[CR22] Johnson MK (1969). The delayed neurotoxic effect of some organophosphorus compounds. Identification of the phosphorylation site as an esterase. Biochem J.

[CR23] Karki P, Ansari JA, Bhandary S, Koirala S (2004). Cardiac and electrocardiographical manifestations of acute organophosphate poisoning. Singap Med J.

[CR24] Langston JL, Myers TM (2011). Diet composition modifies the toxicity of repeated soman exposure in rats. Neurotoxicology.

[CR25] Lei R (2008). Integrated metabolomic analysis of the nano-sized copper particle-induced hepatotoxicity and nephrotoxicity in rats: a rapid in vivo screening method for nanotoxicity. Toxicol Appl Pharmacol.

[CR26] Lockridge O, Schopfer LM (2010). Review of tyrosine and lysine as new motifs for organophosphate binding to proteins that have no active site serine. Chem-Biol Interact.

[CR27] Ludomirsky A (1982). Q-T prolongation and polymorphous (“Torsade de Pointes”) ventricular arrhythmias associated with organophosphorus insecticide poisoning. Am J Cardiol.

[CR28] Massicotte C, Knight K, Van Der Schyf CJ, Jortner BS, Ehrich M (2005). Effects of organophosphorus compounds on ATP production and mitochondrial integrity in cultured cells. Neurotox Res.

[CR29] Maxwell DM, Brecht KM, Koplovitz I, Sweeney RE (2006). Acetylcholinesterase inhibition: does it explain the toxicity of organophosphorus compounds?. Arch Toxicol.

[CR30] McBride EM (2019). Rapid liquid chromatography tandem mass spectrometry method for targeted quantitation of human performance metabolites in saliva. J Chromatogr A.

[CR31] Meade ML (2015). Quantitative proteomic analysis of the brainstem following lethal sarin exposure. Brain Res.

[CR32] Minton NA, Murray VS (1988). A review of organophosphate poisoning. Med Toxicol Advers Drug Exp.

[CR33] Müller U, Krieglstein J (1995). Inhibitors of lipid peroxidation protect cultured neurons against cyanide-induced injury. Brain Res.

[CR34] Myers TM, Langston JL (2011). Diet composition exacerbates or attenuates soman toxicity in rats: implied metabolic control of nerve agent toxicity. Neurotoxicology.

[CR35] Nirujogi RS (2015). Phosphoproteomic analysis reveals compensatory effects in the piriform cortex of VX nerve agent exposed rats. Proteomics.

[CR36] Nomura DK, Casida JE (2011). Activity-based protein profiling of organophosphorus and thiocarbamate pesticides reveals multiple serine hydrolase targets in mouse brain. J Agric Food Chem.

[CR37] Olson H (2000). Concordance of the toxicity of pharmaceuticals in humans and in animals. Regul Toxicol Pharmacol.

[CR38] Peter JV, Sudarsan TI, Moran JL (2014). Clinical features of organophosphate poisoning: a review of different classification systems and approaches. Indian J Crit Care Med.

[CR39] Petroianu G (2015). History of organophosphorus cholinesterase inhibitors and reactivators. Mil Med Sci Lett.

[CR40] Pluskal T, Castillo S, Villar-Briones A, Orešič M (2010). MZmine 2: modular framework for processing, visualizing, and analyzing mass spectrometry-based molecular profile data. BMC Bioinform.

[CR41] Satpute RM, Hariharakrishnan J, Bhattacharya R (2010). Effect of alpha-ketoglutarate and *N*-acetyl cysteine on cyanide-induced oxidative stress mediated cell death in PC12 cells. Toxicol Ind Health.

[CR42] Solomonson LP, Vennesland B, Conn EE, Knowles CJ, Westley J, Wissing F (1983). Cyanide in biology: cyanide as a metabolic inhibitor. Cyanide in biology.

[CR43] Speers AE, Cravatt BF (2004). Profiling enzyme activities in vivo using click chemistry methods. Cell Chem Biol.

[CR44] Suemizu H, Sota S, Kuronuma M, Shimizu M, Yamazaki H (2014). Pharmacokinetics and effects on serum cholinesterase activities of organophosphorus pesticides acephate and chlorpyrifos in chimeric mice transplanted with human hepatocytes. Regul Toxicol Pharmacol.

[CR45] Suemizu H, Kawai K, Murayama N, Nakamura M, Yamazaki H (2018). Chimeric mice with humanized liver as a model for testing organophosphate and carbamate pesticide exposure. Pest Manag Sci.

[CR46] Taira K, Aoyama Y, Kawamata M (2006). Long QT and ST-T change associated with organophosphate exposure by aerial spray. Environ Toxicol Pharmacol.

[CR47] Tuin AW (2009). Activity-based protein profiling reveals broad reactivity of the nerve agent sarin. Chem Res Toxicol.

[CR48] van Helden HPM, van der Wiel HJ, Wolthuis OL (1983). Therapy of organophosphate poisoning: the marmoset as a model for man. Br J Pharmacol.

[CR49] Vizcaíno JA (2014). ProteomeXchange provides globally coordinated proteomics data submission and dissemination. Nat Biotechnol.

[CR50] Vizcaíno JA (2015). 2016 Update of the PRIDE database and its related tools. Nucleic Acids Res.

[CR51] Wang H-P (2009). Metabolic profiles of serum from rats after subchronic exposure to chlorpyrifos and carbaryl. Chem Res Toxicol.

[CR52] Worek F, Mast U, Kiderlen D, Diepold C, Eyer P (1999). Improved determination of acetylcholinesterase activity in human whole blood. Clin Chim Acta.

[CR53] Zdzisińska B, Żurek A, Kandefer-Szerszeń M (2017). Alpha-ketoglutarate as a molecule with pleiotropic activity: well-known and novel possibilities of therapeutic use. Arch Immunol Ther Exp.

[CR54] Zhang L (2010). Systems responses of rats to aflatoxin B1 exposure revealed with metabonomic changes in multiple biological matrices. J Proteome Res.

[CR55] Zhang P (2019). Nanoscavenger provides long-term prophylactic protection against nerve agents in rodents. Sci Transl Med.

