# Supplementary material for: Discovery of treatment for nerve agents targeting a new metabolic pathway
Source: Arch Toxicol. 2020 Jul 27;94(9):3249–64. doi: 10.1007/s00204-020-02820-4 (PMC7415758; doi:10.1007/s00204-020-02820-4)
Supplement: Supplementary file 1 — Supplementary file1 (DOCX 473 kb) [file 204_2020_2820_MOESM1_ESM.docx]

**Discovery of treatment for nerve agents targeting a new metabolic pathway**

Trevor Glaros^1†*^, Elizabeth S. Dhummakupt^1^, Gabrielle M. Rizzo^2^, Ethan McBride^1,3^, Daniel O. Carmany^2^, Linnzi K.M. Wright^4^, Jeffry S. Forster^4^, Julie A. Renner^4^, Ruth W Moretz^4^, Russell Dorsey^4^, Mark R. Marten^6^, Walker Huso^6^, Alexander Doan^6^, Carrie D. Dorsey^5^, Christopher Phillips^4^, Bernard Benton^4^, Phillip M. Mach^1*^

^1^Research and Technology Directorate, BioSciences Division, Combat Capabilities Development Command (CCDC) Chemical Biological Center, 5183 Blackhawk Rd., Aberdeen Proving Ground, MD 21010, USA

^2^Excet, Inc. 6225 Brandon Ave, Suite 360, Springfield, VA 22150, USA.

^3^National Academies of Sciences, Engineering, and Medicine, NRC Research Associateship Programs, 500 Fifth Street, NW, Washington, DC 20001

^4^Research and Technology Directorate, Toxicology & Obscurants Division, Combat Capabilities Development Command (CCDC) Chemical Biological Center, 5183 Blackhawk Rd., Aberdeen Proving Ground, MD 21010, USA

^5^Kirk U.S. Army Health Clinic, 6455 Machine Rd., Aberdeen Proving Ground, MD 21005, USA

^6^Department of Chemical, Biochemical and Environmental Engineering, University of Maryland, Baltimore County (UMBC), Engineering Building, Baltimore, Maryland, USA

***Corresponding Authors:**

Trevor Glaros, PO Box 1663, SM30, Mailstop E529, Los Alamos National Laboratory, Los Alamos, NM 87545, 540-425-6462, tglaros@lanl.gov

Phillip M Mach, 5183 Blackhawk Rd, Building E3150, Gunpowder, MD 21010, 410-417-4527,

phillip.m.mach.civ@mail.mil

† Present address: BioSciences Division, B11 Bioenergy & Biome Sciences, PO Box 1663, SM30, Mailstop E529, Los Alamos National Laboratory, Los Alamos, NM 87545

**SUPPLEMENTAL MATERIAL**

**METHODS:**

*Animal Exposures- Establishing LD_50_*

The median lethal dose (LD_50_), as well as the median effective dose (ED_50_) for moderate toxic signs, associated with intravenous exposure to VX was determined for hairless guinea pigs using an up-and-down procedure^1,2^. Six hairless guinea pigs weighing 345 ± 18 g (average ± standard deviation) were intravenously exposed one at a time to doses of VX ranging from 3.7 to 21.6 µg/kg. The maximum injection volume was 0.5 mL/kg. Toxic signs (Table S1) were continuously monitored for the first 2 h post-exposure, and the onset of each sign was recorded. Lethality was assessed at 24 h post-exposure, and survivors were euthanized with the intravenous administration of a barbiturate euthanasia solution (390 mg/mL sodium pentobarbital). Using the AOT425StatPgm available from the U.S. Environmental Protection Agency (https://www.epa.gov/sites/production/files/2015-06/aot425setup_0.exe) and an assumed sigma of 0.10, the 24 h LD_50_ for intravenous exposure to VX was determined to be 12.1 µg/kg with a 95% confidence limit of 7.3 to 22.1 µg/kg. The ED_50_ for moderate toxic signs was determined to be 4.1 µg/kg with a 95% confidence limit of 3.7 to 4.3 µg/kg.

Table S1. Ethogram of the behavioral toxic signs associated with intravenous exposure to VX for hairless guinea pigs.

| **Category** | **Toxic Sign** | **Description** |
| --- | --- | --- |
| Mild | Ataxia | Inability to coordinate muscle movements |
|  | Exopthalmus | Abnormal protrusion of the eyeballs |
|  | Salivation | Secretion of saliva |
| Moderate | Tearing | Secretion of milky white tears |
|  | Muscle Fasciculation | A small, local, involuntary muscle contraction |
|  | Tremor | Involuntary trembling or quivering; uncontrolled muscle activity |
|  | Subjerking | Uncontrolled jerking of the head |
| Severe | Collapse | Unable to support body weight but head can still be held up |
|  | Convulsion | Violent, involuntary muscle contraction or a series of muscle contractions |
|  | Gasping | Laborious or convulsive breathing |
|  | Prostrate | Extreme exhaustion and powerlessness coupled with inability to hold head up and loss of righting reflex |

*Proteomics*

Sample preparation. Plasma samples were prepared as described previously (Tran et al., 2018). Briefly, frozen plasma samples were thawed on ice and quantitated for total protein concentration using a BCA protein assay kit (Pierce, Rockford, Illinois). A 150 µg aliquot of each sample was removed for digestion and added to respective tubes of 200 µl 50mM triethylammonium bicarbonate (TEAB). The samples were then denatured by adding 300 µl 10M urea and 10 µl 1M dithiothreitol; they were heated at 56°C for 30 min while shaking. Samples were allowed to cool and were alkylated by adding 40 µl of 0.5M iodoacetamide and incubated at room temperature in the dark for 30 min. Following alkylation, 900 µl of 50mM TEAB was added to each sample to bring the urea concentration down to ~2 M. 4 µg of Trypsin/Lys-C (Promega, Madison, Wisconsin), resuspended to 1 µg/µl in 50 mM TEAB, was added to each sample. Digestion occurred overnight at 37°C with shaking. Digestion was terminated by adding 15 µl of 100% formic acid using a glass syringe. Oasis HLB 1 cc (30 mg) reverse phase cartridges (Waters, Milford, Massachusetts) were used to desalt each sample following the manufacturer’s protocol. Eluted samples were dried in a speed-vac overnight. Each dried sample was resuspended to a final concentration of 2 mg/mL (using concentrations determined in BCA assay) in 30% acetonitrile. A “MasterMix” sample was then created by combining 3 µl of each of the 54 samples. Peptide quantification was then carried out on all samples, including the MasterMix, using a Pierce Quantitative Colorimetric Peptide Assay (Pierce, Rockford, Illinois). 30 µg of each sample was removed (with six 30 µg aliquots removed from the MasterMix) and 30% acetonitrile was added to each aliquot to bring the volume up to 45 µl. 5 µl of 1 M TEAB was then added to each aliquot to bring the final volume up to 50 µl.

TMT labeling. The 30 µg/50 µl aliquots were TMT-labeled using a Thermo Scientific 5 mg TMT 10plex kit (Pierce, Rockfordd, Illinois) according to the manufacturer’s instructions. Briefly, the kit reagents were brought to room temperature, spun down at 15,000 rpm for 3 min, and each TMT vial was resuspended in 256 µl DriSolv® acetonitrile. Vials were vortexed for 5 min and spun down briefly. 20 µl of each tag was then added to its corresponding sample (see Table S2), and the labeling scheme was repeated identically for all 6 animals for a total of 6 10plexes, with each 10plex including one of the identical 30 µg/50 µl MasterMix aliquots. After adding the tags, the samples shook at room temperature for 1 hour. Reactions were then quenched by adding 4 µl of 5% hydroxylamine (diluted from 50% in 0.1 M TEAB) and shook for an additional 15 min at room temperature. Samples were then pooled together based on animal, with one MasterMix per pool, for a total of six TMT pools. All pools were dried in the speed-vac.

Basic reverse phase liquid chromatography (bRPLC). Each of the dried TMT-labeled peptide pools were fractionated by bRPLC as described in Keshishian et al. (2015), with some modifications. Briefly, each pool was reconstituted in 100 µl 100% acetonitrile, vortexed, and incubated at 37°C for 5 min. 100 µl of 20 mM ammonium formate/10% acetonitrile pH 10 (buffer A) was then added to the pool which was again vortexed and incubated at 37°C for 5 min. Finally, another 800 µl of buffer A was added to the pool which was vortexed for 2 min, and spun down in a centrifuge at max speed for 3 min. The sample pool was loaded by syringe pump onto a Waters XBridge C18 5 µm 4.6x250mm column at a rate of 6 mL/hour. Then the column was connected to an Agilent 1260 HPLC pump system (Santa Clara, California) equipped with an analytical-scale fraction collector. The flow rate was set to 200 ml/min. Buffer A was made up of 20 mM ammonium formate/10% acetonitrile pH 10, and Buffer B was made up of 20 mM ammonium formate/90% acetonitrile pH 10. Peptides were separated using the following gradient: 0–5 min: 0% B, 5–13 min: 0%–15% B, 13–46 min: 15%–28.5% B, 46–51.5 min: 28.5%–34% B, and 51.5–64.5 min: 34%–60% B. Samples eluted in the first 5.5 min were pooled into 3 “start” fractions and the final 9.2 min were pooled into 5 “end” fractions. Between the “start” and “end” fractions, single fractions were collected every 0.6 min for a total of 84 fractions. The 84 fractions were concatenated into 14 fractions, designated F1-F14, and all fractions were acidified with 10% formic acid (100 µl for each of the “start” and “end” fractions, 200 µl for each of the F1-F14 fractions) and dried in a speed-vac.

Liquid chromatography tandem mass spectrometry analysis (LC-MS/MS). Each pool was processed separately via LC-MS/MS analysis. Immediately prior to analysis, each fraction was reconstituted in 95% water/5% acetonitrile with 0.1% formic acid (20 µl for each “start” and “end” fraction, 40 µl for each F1-F14 fraction) and vortexed for 2 min to completely dissolve the sample. Samples were then centrifuged at max speed for 5 min and transferred to respective autosampler vials (18 µl transferred for “start” and “end” fractions, 38 µl transferred for F1-F14 fractions). The 3 “start” fractions were combined into a single vial, and the 5 “end” fractions were combined into another vial. Each fraction was analyzed on a Thermo Fisher QExactive Plus mass spectrometer coupled to a Dionex Ultimate 3000 UHPLC (Thermo Fisher Scientific). 2 µl injections of each sample were pre-concentrated on a reverse-phase trapping column (300 µm i.d. x 5mm C18 PepMap100, 5µm, 100Å, Thermo Fisher) and then resolved on a 75 µm x 50cm EASY-Spray column packed with PepMap RSLC, C18, 2 µm, 100Å particles (Thermo Fisher) using a 182 min multistep gradient [0–150 min: 5%–35% B, 150–158 min: 35%– 60% B, 158–161 min: 60%–90% B, 161–171 min: 90% B hold, 171– 172 min: 90% B, and 172–182 min: 5% B hold]. For the gradient, the A buffer is 100% H2O/0.1% formic acid and the B buffer is 98% acetonitrile/ 2% H2O/0.1% formic acid. Full MS scans were acquired at a resolution of 70,000. The top 12 precursors were selected for MS2 data-dependent fragmentation. ddMS2 scans were acquired using the Orbitrap at a resolution of 35,000, with a scan range of m/z 300-1700 in profile mode, with the first mass fixed to m/z 100. Monoisotopic precursor selector was set to “on” and charge state filter was set to exclude charges of 1, 8, and >8. Higher-energy collisional dissociation (HCD) was used to generate MS2 spectra with normalized collision energy (NCE) set to 26%. AGC target was set to 3e6 for MS1 and 1e5 for MS2. The maximum injection time was 50 ms for MS1 and 120 ms for MS2. Dynamic exclusion was set for 20 s with a 5 ppm mass window.

Data Processing. Intensities of MS2-generated reporter ions were used for quantification using PEAKS X software (Bioinformatics Solutions Inc., Waterloo, ON, Canada) against UniProt database for Cavia porcellus. Data refinement settings were set to correct precursor [DDA] (mass only), associate feature with chimera scan, and to filter features to only keep charges between 2 and 8. Identification settings were set to a parent mass error tolerance of 10 ppm (using monoisotopic mass) with fragment mass error tolerance of 0.02 Da. Trypsin was selected as the enzyme, with semispecific digest mode and a maximum of 1 missed cleavage per peptide. TMT 10plex (229.16 Da) and carbamidomethylation (57.02 Da) were set as fixed PTMs and oxidation (15.99 Da on methionine) was set as a variable PTM, with a maximum of 2 variable PTMs allowed per peptide. Purity corrections were set in the Quantification node using the lot-specific purity corrections of the TMT kit. Quantification settings were also set to a mass error tolerance of 0.02 Da with MS2 reporter ion type and FDR threshold of 1%. For quantification, inter-experiment normalization was performed using the -131 MasterMix channel as the spiked channel, with auto normalization selected. Spectrum filter settings in the quantification node were set to FDR of 1% and quality score of ≥15 with the reference channel present (with the -126 base/pre-exposure channel used as reference). Results were filtered to include proteins with at least 1 unique peptide and fold-change of 2 or greater. PCA plot and heatmap were both generated in Perseus version 1.5.5.3 using protein abundances exported from PEAKS X. Heatmap was generated by averaging time point groups.

Statistical Analysis: Multivariate Adaptive Regression Splines (MARS). For the MARS analysis the raw intensity values were transformed into log2-fold changes to normalize the data across the biological replicates. A MARS model was then fitted to the fold changes across all the replicates for each identified biomarker (6 replicates, 9 time points) using the ARESLab package in Matlab. The ARESpredict function was used with default settings to determine the quality of fit using mean square error of the actual data and 10,000 random permutations of the fold change levels. The p-value was assessed by calculating the fraction of randomly permutated models with a better fit than the actual model. If the p-value was less than 0.05, the site was determined to be dynamic over the time course.

**Table S2: TMT Labeling Scheme**

*Repeated identically for each animal pool.

| **Tag** | **Sample** |
| --- | --- |
| 126 | Base (pre-exposure) |
| 127N | 1 hour |
| 127C | 6 hour |
| 128N | 24 hour |
| 128C | 48 hour |
| 129N | 4 day |
| 129C | 7 day |
| 130N | 10 day |
| 130C | 14 day |
| 131 | MasterMix |

*Metabolomics*

Sample preparation. A 50 ml of thawed plasma was mixed with 410 ml of extraction solution containing isotopically labelled internal standard (ISTD) mixture. The extraction solution was made fresh in sufficient quantities to perform extraction of all samples at once. It was composed of 400 parts of a precipitation solution (8:1:1 acetonitrile: methanol: acetone) and 10 parts of the ISTD mixture (working stock). The ISTD mixture is prepared by making working stocks of each solution at 2 mg/ml by dissolving 10 mg of each standard in 5 ml of 90:10 water: acetonitrile. A working stock solution was prepared by combining the following volumes of each ISTD stock into a single vial containing 4715 ml of Fisher Optima gold label water with 0.1% FA (final volume 5000 ml): d3-creatine (10 ml), d10-leucine (10 ml), d3-L-tryptophan (10 ml), 13C6-citric acid (20 ml), 13C11-tryptophan (100 ml), 13C6-leucine (10 ml), 13C6-L-phenylalanine (10 ml), T-BOC-L-tertleucine (10 ml), and T-BOC-L-aspartic acid (5 ml). Upon addition of the extraction solution, each sample is vortexed and stored at 4C for 60 min to complete protein precipitation. Each sample is centrifuged at 20 000 g for 10 min at 4C to pellet precipitate. A 375 ml of supernatant is transferred to a new tube taking care not to disturb the protein pellet. Each sample is dried to completeness and stored at -80C until LC-MS analysis.

Liquid chromatography mass spectrometry analysis (LC-MS). Immediately prior to LC-MS analysis each fraction was reconstituted in 50 ml of Fisher Optima gold label H2O with 0.1% FA and vortexed briefly. Samples were placed in the refrigerator for 10–15 min to allow for resuspension. Finally, each sample is centrifuged at 20 000 g for 10 min and transferred into glass autosampler vials (Agilent, Santa Clara, California) for analysis. Each sample was analyzed on a Thermo Fisher Orbitrap Q Exactive Plus mass spectrometer coupled to a Thermo Fisher Ultimate 3000 analytical system. Injections of each sample (2 ml) were resolved with the analytical pump (350 ml/min) on a 100 mm 2.1 mm id ACE Excel 1.7 C18-PFP (Mac-Mod Analytical) using a 22.5 min flow gradient [0!3 min at 100% A, ramp from 3 ! 13 min at 20% A/80% B, hold 13 ! 16 min at 20% A/80% B, return to initial conditions 16 ! 20 min, hold 20 ! 22.5 min at 100% A, curve ¼ 5]. For the flow gradient, the A buffer is water with 0.1% formic acid and the B buffer is 100% acetonitrile. Orbitrap MS1 scans were acquired with a resolution of 70 000 with a scan range of m/z 70–1000. AGC target was set to 3E6 with a maximum injection time of 100 ms. All metabolomics data were acquired in positive and negative ionization mode using the heated electrospray ionization source (HEIS). The source settings were as follows: spray voltage: 63.7 kV, capillary temperature: 325C, sheath gas (N2): 30 arbitrary units (AU), auxiliary gas (N2): 10 AU, and the probe heater: 350C.

**Table S3:**

Table for signs and symptoms over time for the 0.4 LD50 animals used in this study.


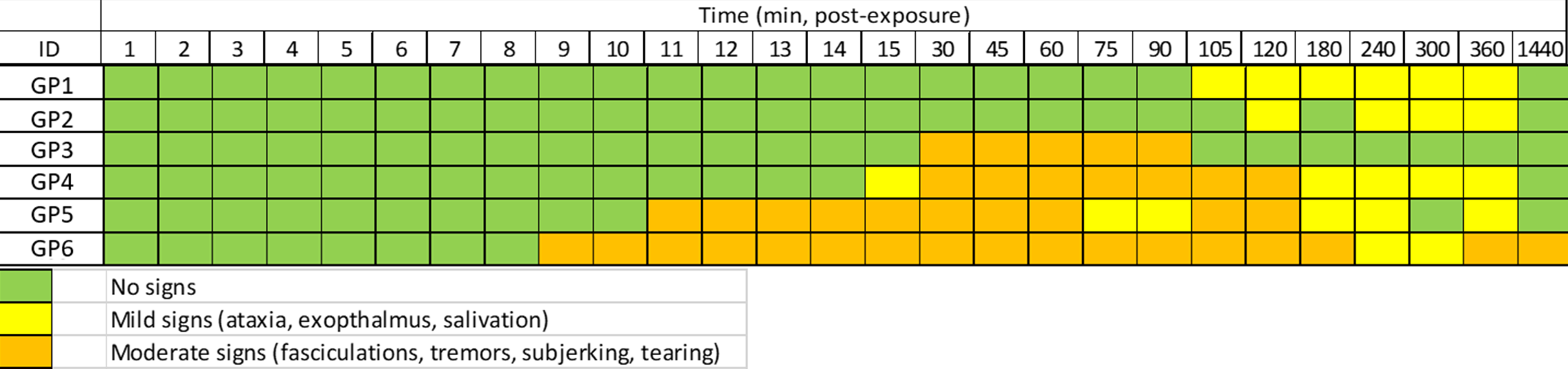


**Table S4: 72 Significant Proteins (p<0.05) Determined by MARS Analysis**

| **Description** | **p_values** |
| --- | --- |
| apolipoprotein C-III precursor | 0.0259 |
| catalase | 0.0009 |
| alpha-enolase | 0.0046 |
| immunoglobulin J chain precursor | 0.0219 |
| angiopoietin-related protein 6 | 0.0212 |
| flavin reductase (NADPH) | 0.0066 |
| heat shock protein HSP 90-alpha | 0.0282 |
| plexin domain-containing protein 2 | 0.0452 |
| thrombospondin-4 | 0.0309 |
| creatine kinase M-type | 0.0031 |
| hemoglobin subunit beta | 0.0051 |
| hemopexin | 0.0493 |
| pantetheinase | 0.0468 |
| beta-enolase | 0.0209 |
| acetyl-CoA acetyltransferase cytosolic | 0.0128 |
| proteasome subunit beta type-1 | 0.0226 |
| cytosolic non-specific dipeptidase | 0.0015 |
| xaa-Pro dipeptidase | 0.0010 |
| malate dehydrogenase cytoplasmic | 0.0072 |
| adenosylhomocysteinase | 0.0161 |
| fructose-1 6-bisphosphatase 1 | 0.0043 |
| alpha-2-antiplasmin isoform X2 | 0.0232 |
| fructose-bisphosphate aldolase C | 0.0491 |
| mast/stem cell growth factor receptor | 0.0165 |
| liver carboxylesterase 1-like | 0.0195 |
| nidogen-1 | 0.0102 |
| actin alpha skeletal muscle | 0.0022 |
| isocitrate dehydrogenase cytoplasmic | 0.0398 |
| C4b-binding protein beta chain | 0.0226 |
| serum paraoxonase/arylesterase 1 isoform X1 | 0.0494 |
| actin alpha cardiac muscle 1 | 0.0013 |
| plexin-C1 | 0.0183 |
| serotransferrin | 0.0359 |
| lactotransferrin | 0.0441 |
| adiponectin | 0.0262 |
| macrophage colony-stimulating factor 1 receptor | 0.0207 |
| hemoglobin subunit alpha | 0.0204 |
| prolyl endopeptidase FAP | 0.0129 |
| collectin-10 | 0.0175 |
| gamma-glutamyl hydrolase | 0.0320 |
| carbonic anhydrase 1 isoform X1 | 0.0219 |
| cytosolic non-specific dipeptidase | 0.0012 |
| alpha-enolase isoform X1 | 0.0058 |
| insulin-like growth factor I isoform X1 | 0.0088 |
| CD109 antigen | 0.0268 |
| angiopoietin-related protein 6 | 0.0213 |
| CD44 antigen isoform X1 | 0.0371 |
| CD44 antigen isoform X2 | 0.0398 |
| CD44 antigen isoform X3 | 0.0401 |
| CD44 antigen isoform X4 | 0.0405 |
| CD44 antigen isoform X5 | 0.0406 |
| CD44 antigen isoform X6 | 0.0396 |
| CD44 antigen isoform X7 | 0.0413 |
| CD44 antigen isoform X8 | 0.0381 |
| cytosolic non-specific dipeptidase | 0.0011 |
| cytochrome b5 | 0.0001 |
| beta-mannosidase | 0.0387 |
| alpha-2-antiplasmin isoform X1 | 0.0221 |
| apolipoprotein C-III isoform X1 | 0.0254 |
| apolipoprotein A-I | 0.0445 |
| CD97 antigen isoform X1 | 0.0014 |
| CD97 antigen isoform X2 | 0.0019 |
| CD97 antigen isoform X3 | 0.0019 |
| CD97 antigen isoform X4 | 0.0025 |
| C4b-binding protein beta chain | 0.0216 |
| serum paraoxonase/arylesterase 1 isoform X2 | 0.0491 |
| angiopoietin-related protein 6 | 0.0203 |
| creatine kinase M-type | 0.0020 |
| microfibril-associated glycoprotein 4 | 0.0181 |
| cytosolic non-specific dipeptidase | 0.0012 |
| uncharacterized protein LOC101787890 | 0.0037 |
| alpha-fetoprotein | 0.0001 |
|  |  |

**REFERENCES:**

1.Dixon WJ, Mood AM (1948) A method for obtaining and analyzing sensitivity data. J Am Stat Assoc 43:109-26.

2. Rispin A, Farrar D, Margosches E, Gupta K, Stitzel K, Carr G, Greene M, Meyer W, McCall D (2002) Alternative methods for the median lethal dose (LD50) test: the up-and-down procedure for acute oral toxicity. ILAR J 43:233-43.
